# Medical Students’ Perception Toward Various Human Anatomy Teaching Methods in Khartoum, Sudan

**DOI:** 10.7759/cureus.96954

**Published:** 2025-11-16

**Authors:** Ahmed Aydrose, Hussein Elsdaig, Adnan Abdalla, Mohamed Khalafalla Adlan Abdelsadig, Duaa Elderderi

**Affiliations:** 1 Surgery, The National Ribat University, Khartoum, SDN; 2 Surgery, Sudan Medical Specialization Board, Khartoum, SDN; 3 General Internal Medicine, The National Ribat University, Khartoum, SDN; 4 Orthopaedics, The National Ribat University, Khartoum, SDN; 5 Orthopaedics, University Hospital Waterford, Waterford, IRL

**Keywords:** medical and clinical anatomy, medical education, students’ perceptions, sudan, teaching methods

## Abstract

Background

Anatomy teaching uses both traditional and modern methods. Although cadaveric dissection remains the gold standard, students face challenges in visualizing embryological processes and applying histological knowledge clinically. Prior studies have mainly focused on Western medical schools, overlooking resource-limited settings such as Sudan. This study aimed to explore students’ perceptions of anatomy teaching methods in such a setting.

Methodology

A cross-sectional survey was conducted among second to fifth-year medical students who had completed at least half of their anatomy syllabus. Data were collected using a structured questionnaire and analyzed using SPSS version 29 (IBM Corp., Armonk, NY, USA).

Results

A total of 248 students participated in this study, with a response rate of 77.0%; 63.7% of the participants were female. Overall, 43% favored lecture-based teaching. For practical sessions, the most preferred approaches were labeled structures combined with on-body teaching (84.7%), dissected bodies (48.4%), anatomical models (39.1%), and multimedia resources (34.7%). Reported challenges in embryology included difficulty understanding developmental sequences, limited time, and visualization difficulties (52.4%), while histology challenges centered on insufficient lecture (33.9%) and practical (33.1%) time. Gender comparisons showed no significant differences in preferences, challenges, or proposed solutions (p > 0.05).

Conclusions

Students favor a blended approach that combines traditional cadaveric dissection with modern visual tools, clinical integration, and increased time for histology and embryology.

## Introduction

Anatomy is a cornerstone of medical education, providing the essential foundation for interpreting clinical symptoms, performing physical examinations, and ensuring safe clinical practice [1,2]. A comprehensive anatomy curriculum typically covers gross anatomy, embryology, and histology, using diverse teaching approaches. Traditional methods include didactic lectures, cadaveric dissection, prosection, and anatomical models, while modern strategies incorporate computer-assisted learning, virtual and augmented reality, radiological imaging, problem-based learning (PBL), and integrated teaching [3]. Innovative teaching strategies such as student-selected modules, team-based learning, and flipped classrooms have gained attention for promoting active learning and teamwork skills. Evidence shows that such learner-centered approaches enhance knowledge retention and satisfaction compared to traditional lectures, particularly when integrated with a clinical context [4].

Anatomy education has historically relied on cadaveric dissection, evolving over centuries to include new techniques such as PBL, three-dimensional (3D) visualization, and e-learning platforms [5,6]. Cadaveric dissection remains highly valued for providing tactile and spatial understanding [7], but studies show that students increasingly benefit from blended approaches combining traditional and modern tools [8,9]. Recent reforms in anatomy curricula worldwide emphasize clinical integration and learner-centered methods [10,11]. However, resource-limited settings often face constraints in adopting advanced modalities, making lectures and cadaver-based instruction the dominant methods [12]. Most previous studies have been descriptive, focusing on student satisfaction without assessing learning outcomes or long-term retention. Furthermore, research from high-income countries often assumes access to advanced digital tools, limiting applicability to resource-limited settings. Few studies have systematically compared blended versus traditional models in such contexts, which indicates the need for evidence from low-resource institutions like those in Sudan.

In Sudan, anatomy remains a pivotal component of medical training, but its delivery faces significant challenges [13-15]. Limited infrastructure, financial constraints, and scarcity of advanced teaching tools restrict innovation [13]. Ongoing sociopolitical conflict has further disrupted education, forcing institutions to relocate or adapt under resource-limited conditions. These circumstances highlight the need to understand how students perceive anatomy teaching methods in such contexts.

Despite its centrality, anatomy is often perceived as abstract and content-heavy, particularly by first-year students. Challenges include difficulty connecting theory to clinical application, a lack of visualization tools for embryology and histology, and limited time in dissection laboratories. These barriers can lead to frustration, disengagement, and reduced academic performance [16,17]. Although previous studies in other countries have evaluated students’ attitudes toward teaching methods, little research has been conducted in Sudan, leaving an important gap in understanding [14,15].

Recent studies have highlighted the growing integration of virtual dissection tables, mixed reality, and flipped classroom models in anatomy education, which have been shown to improve spatial understanding and learner engagement [17].

Despite its importance, there is little evidence on how anatomy is taught and perceived in Sudanese medical schools. This study addresses that gap by systematically examining students’ perceptions within a low-resource academic setting. Findings aim to inform curriculum reform and guide future adoption of blended or digitally enhanced teaching approaches suited to local constraints.

The general objective is to explore medical students’ perceptions of teaching methods in human anatomy at the University of Medical Sciences and Technology (UMST). The specific objectives are: (1) assess students’ ratings of different anatomy teaching methods using a structured scale, (2) examine how combined teaching approaches influence perceived understanding, (3) identify major challenges and proposed improvements in embryology and histology learning, and (4) evaluate gender-based differences in preferences and challenges using inferential statistical analysis.

## Materials and methods

Study design

This descriptive, cross-sectional survey was conducted between August and November 2024. Data were collected using a structured questionnaire developed by the authors. The questionnaire was pilot-tested on students to assess clarity and reliability, and content validity was reviewed by anatomy education experts. The 14-item questionnaire comprised mainly closed-ended questions rated on specific answer options (Appendices).

Study area

The study was conducted at the Faculty of Medicine, University of Medical Sciences and Technology (UMST). Originally located in Khartoum, Sudan, UMST was temporarily relocated to Dodoma, Tanzania, due to ongoing conflict. The faculty follows a hybrid anatomy curriculum integrating lectures, tutorials, PBL, and cadaver-based practical sessions. Facilities include dissection laboratories, models, and histology labs. Although the relocation ensured continuity of teaching, it may have influenced students’ perceptions due to changes in infrastructure and learning environment, an aspect considered among the study’s limitations. The curriculum covers gross anatomy of the head and neck, thorax, abdomen, back, and limbs over a period of two and a half years. Instruction is delivered mainly through lectures, which account for about 50% of theoretical hours, supplemented by tutorials and PBL sessions (25%), and practical sessions in small groups (25%). Practical sessions include rotations between cadavers, prosections, and models. Embryology is taught during the first two years, primarily through didactic lectures supported by diagrams, charts, and videos, although the use of advanced digital tools such as animations and 3D embryological models remains limited due to infrastructural constraints. Histology is delivered through lectures and laboratory sessions using light microscopy, while virtual microscopy and digital histological atlases are still under development.

Study population

The study population included all medical students who had completed at least one year of anatomy instruction (N = 322). A total of 248 students completed the questionnaire, yielding a response rate of 77.0%. Participation was voluntary, and no incentives were provided. Incomplete responses were excluded before analysis. To prevent multiple submissions, the Google Forms “limit to one response per user” feature was enabled. The study included students from the second to fifth academic years.

Inclusion criteria

Students were eligible to participate if they had completed their first year of medical school and provided informed consent.

Sampling technique

A census sampling strategy was employed, including all eligible students from the second to fifth academic years. This approach minimized selection bias and ensured comprehensive coverage of students’ perceptions.

Data collection

Data were collected using a structured questionnaire adapted from Swetha and Thenmozhi (2020) [18] and modified for the local context. The instrument consisted of 18 items covering teaching methods in lectures, tutorials, and practical sessions; preferred learning aids; challenges in embryology and histology; guidance during dissection; assessment methods; and suggested solutions. The questionnaire was distributed electronically via Google Forms, and students completed it independently and anonymously.

Data analysis

Data were entered, cleaned, and analyzed using SPSS version 29 (2022) (IBM Corp., Armonk, NY, USA). Incomplete responses were excluded before analysis. Descriptive statistics (frequencies and percentages) summarized responses. Associations between categorical variables, such as gender and preferred teaching modality, were assessed using Pearson’s chi-square test, after confirming that assumptions for expected cell counts were met. Reliability and validity were supported by the use of a previously published and adapted questionnaire. Multivariate analyses were not performed, as the primary objective was to provide a descriptive overview of students’ perceptions.

Ethical approval

Ethical approval was obtained from the Faculty of Graduate Studies, UMST (approval number: UMST/ANAT/IRB/2024/06) and the UMST Anatomy Department. Participation was voluntary, informed consent was obtained, and no personal identifiers were collected.

## Results

**Figure 1:**
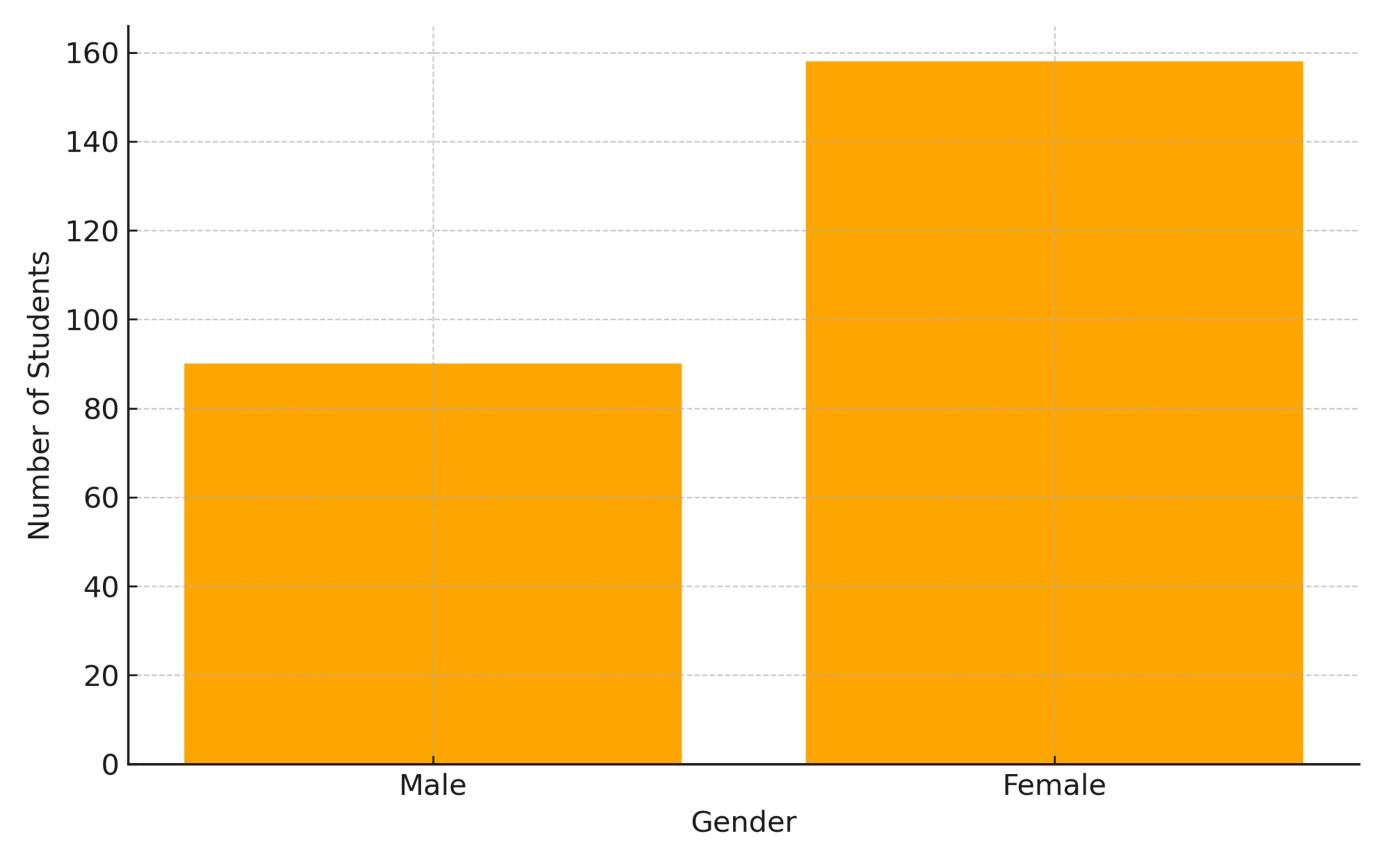
Gender distribution of participants (n = 248).

A total of 248 students completed the questionnaire (response rate = 77.0%), of whom 157 (63.7%) were female and 90 (36.3%) were male (Figure 1).

Overall preferences for teaching modalities

For theory sessions, lectures were most frequently preferred (43.1%), followed by small group discussions (27.0%), interactive media (14.9%), and PBL (14.9%). PowerPoint was preferred by 60.5% of students compared to the chalkboard (39.5%).

For tutorial sessions, anatomical models (39.1%) and multimedia resources (34.7%) were most useful; small group discussions were selected by 22.6%.

For practical sessions, dissected bodies (48.4%) were preferred, followed by anatomical models (29.8%), educational videos (16.1%), and prosections (5.6%). Guidance during dissections was most effective when labeled structures were combined with on-body teaching (84.7%) (Table 1).

**Table 1:** Students’ preferred teaching modalities for theory, tutorials, and practical sessions (n = 248). Data are presented as frequency (%).

Variable	Category	Frequency	Percent (%)
Theory	Lectures	107	43.1
Theory	Small group for discussion	67	27
Theory	Interactive Media	37	14.9
Theory	Problem-based learning	37	14.9
Preferred teaching methods	PowerPoint	150	60.5
Preferred teaching methods	Chalkboard	98	39.5
Tutorial sessions	Anatomical models	97	39.1
Tutorial sessions	Multimedia	86	34.7
Tutorial sessions	Small group for discussion	56	22.6
Tutorial sessions	Repeat the theoretical contents	9	3.6
Practical sessions	Dissected body	120	48.4
Practical sessions	Anatomical models	74	29.8
Practical sessions	Educational video	40	16.1
Practical sessions	Prosection	14	5.6
Preferred guidance methods during dissection sessions	Labeled structures (only)	19	7.7
Preferred guidance methods during dissection sessions	Labeled structures + teaching on the body	210	84.7
Preferred guidance methods during dissection sessions	Teaching on dissected body (only)	19	7.7

Challenges in embryology and histology

Students reported challenges in embryology, including difficulty visualizing developmental sequences, inadequate teaching time, and poor visualization of complex concepts. Histology challenges included insufficient lecture (33.9%), practical time (33.1%), and conceptual difficulty. Suggested solutions included using more visual aids (including 3D), simplifying content, increasing lecture/practical hours, integrating with clinical cases, and repetitive reinforcement (Table 2).

**Table 2:** Challenges and suggested solutions for teaching anatomy (n = 248).

Variable	Challenges	Suggested Solutions
Embryology	Difficulty visualizing development; inadequate time	Use more visual aids (including 3D), simplify content, extend lecture/lab time
Histology	Limited lecture/practical hours; confusing concepts	More teaching time, integration with clinical cases, repeated reinforcement
Practical sessions	Limited time; visualization problems	More time, enhanced use of dissection and visual aids, repetitive reinforcement

Preferred sources of study materials

**Figure 2:**
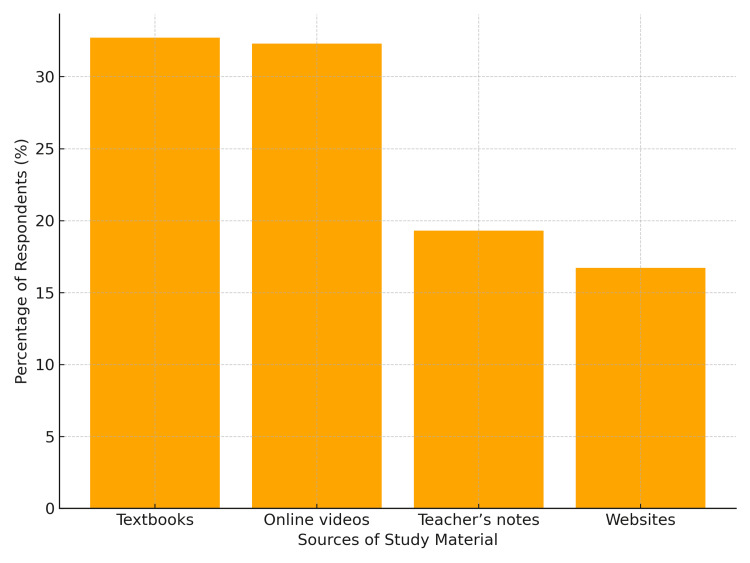
Preferred sources for studying anatomy (n = 248).

Textbooks and online videos were most popular (~one-third of respondents each), followed by teachers’ notes and websites (Figure 2).

**Figure 3:**
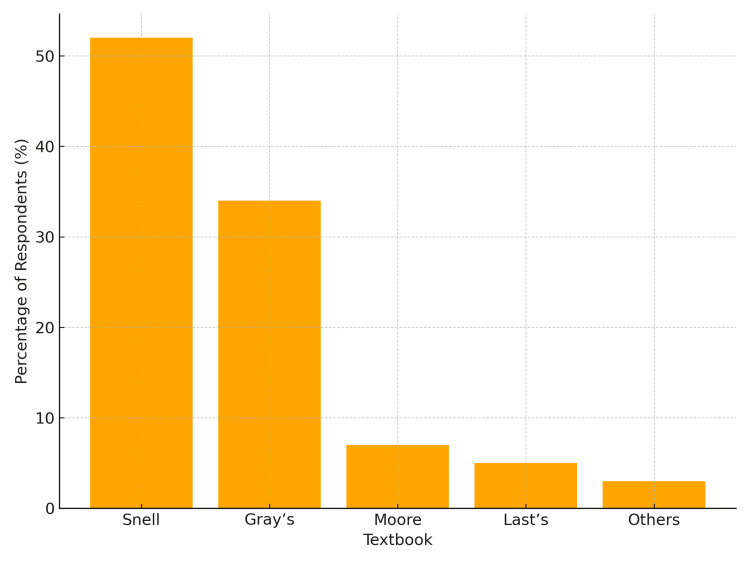
Preferred textbooks for studying anatomy (n = 248).

Regarding textbooks, Snell’s Clinical Anatomy was most frequently preferred, followed by Gray’s Anatomy (Figure 3).

Gender-based analyses

Gender-based differences are summarized in Table 3 and Table 4. No statistically significant differences were observed between male and female students for preferences in theory, practical sessions, tutorials, or study resources (Pearson’s chi-square test, all p > 0.05). Both genders reported similar challenges in embryology and histology, and proposed solutions were comparable. Labeled structures combined with on-body teaching during dissections were preferred by 83.3% of males versus 85.4% of females (p = 0.856). P-values above 0.05 indicate non-significance. Analyses were limited to descriptive and bivariate statistics due to sample size constraints (Table 4).

**Table 3:** Comprehensive gender-based differences in students’ preferences toward the best teaching modality and study resources (n = 248). Statistical test: Pearson’s chi-square; significance threshold: p < 0.05.

Variable	Category	Male (n = 90)	Female (n = 158)	P-value
Best teaching modality for theory	Interactive media	11	26	0.227
	Lecture answers	45	62	
	Problem-based learning	15	22	
	Small group discussion	19	48	
Preferred teaching methods	Chalkboard	32	66	0.336
	PowerPoint	58	92	
Best teaching modality for practical sessions	Anatomical models	26	48	0.952
	Dissected body	44	76	
	Educational video	14	26	
	Prosection	6	8	
Preferred guidance methods during dissection	Labeled structures + on‐body teaching	75	135	0.856
	Labeled structures (only)	8	11	
	Teaching on dissected body (only)	7	12	
Preferred source for studying anatomy	Textbooks	32	58	0.854
	Teacher’s notes	20	28	
	Online videos	31	58	
	Website application	7	14	
Preferred textbook for studying anatomy	Grey’s Anatomy	28	56	0.461
	Snell’s Clinical Anatomy	50	77	
	Moore’s Clinically Oriented Anatomy	7	10	
	Sameh Doss Books	3	3	
	BRS Anatomy	0	2	
	Last’s Anatomy	2	10	

**Table 4:** Comprehensive gender-based differences in students’ perception toward encountered challenges and proposed solutions (n = 248). Data are presented as frequencies (%). Statistical test: Pearson’s chi-square; significance threshold: p < 0.05.

Variable	Category	Male (n = 90)	Female (n = 158)	P-value
Best possible solutions (general)	Additional practical tutorial session	23	34	0.815
	More clinical correlation	22	43	
	More visual aids	17	35	
	Repetitive reinforcement	28	46	
Specific problems in understanding embryology	All options	51	79	0.52
	Inability to understand sequence of events	14	27	
	Inability to visualize	14	22	
	Inadequate time	11	30	
Best possible solutions for embryology and histology	Use more visual aids	28	44	0.501
	More time in lecture	29	42	
	Simplify information	28	57	
	More time in practice	5	15	
Specific problems in understanding histology	Insufficient time in practical sessions	32	50	0.932
	Difficult/Confusing concepts	16	31	
	Lack of audio‐visual aids	12	23	
	Insufficient time in lecture	30	54	
Best possible methods for tutorial sessions	Small group for discussion	19	37	0.354
	Anatomical models	41	56	
	Multimedia	26	60	
	Repeat theoretical contents	4	5	
Best possible solutions for practical sessions	Additional time required	27	51	0.868
	More visual aids (including dissection)	24	44	
	Others	16	30	
	Repetitive reinforcement	23	33	

## Discussion

This study confirms the continuing significance of conventional approaches in anatomy learning, although digital tools are increasingly important. A substantial proportion of students identified lecture-based teaching as the most effective approach for theory sessions, consistent with earlier research emphasizing reliance on systematic content presentation and structured class instruction [14-21]. Additionally, a higher number of students preferred PowerPoint presentations over chalkboard instruction, aligning with Vashishtha et al. (2021), who reported that PowerPoint improves visibility and clarity of anatomical structures [14-21].

Histology and embryology remain challenging for learners, often perceived as abstract and disconnected from practical application. Over half of the respondents reported difficulties in visualizing developmental sequences and understanding complex embryological processes. Limited lecture and practical time were also highlighted as major constraints in histology. These findings echo previous research emphasizing the need for visual aids, curricular adjustments, and improved integration with clinical contexts [22,23]. Local evidence from Sudanese students supports these observations, highlighting similar challenges in anatomy education, particularly in embryology and histology [20].

Our findings demonstrate that visual and tactile resources continue to be valuable for learning. Multimedia tools and anatomical models were highly preferred, reflecting the effectiveness of interactive 3D models and virtual laboratories in enhancing comprehension [14]. Cadaveric dissection remains central: nearly half of the students favored dissected bodies, and most preferred labeled structures combined with on-body teaching. This aligns with previous studies confirming the role of dissection in strengthening knowledge retention and understanding of anatomical relationships [15,16]. Innovations such as plastination offer biosafe alternatives for cadaver preservation [18], and social media platforms are increasingly recognized as supplementary resources in anatomy education [19]. Blended approaches combining traditional and modern methods have been shown to enhance knowledge retention [21]. Prior surveys, including Swetha and Thenmozhi (2020), further validate the use of structured questionnaires for evaluating student perceptions [17].

Despite growing access to digital resources, traditional materials remain central. Textbooks, particularly Snell’s Clinical Anatomy and Gray’s Anatomy, were most frequently used, consistent with studies emphasizing the continued reliance on traditional references even in the digital era [23-27].

Gender-based analysis revealed no statistically significant differences in preferences, challenges, or proposed solutions (p > 0.05). This may reflect equitable teaching impact across genders, cultural uniformity in learning approaches, or methodological constraints such as sample size and response distribution.

Digital inequity emerged as a potential determinant of anatomy education effectiveness in this context. Limited access to virtual tools, reliable internet, or multimedia resources may constrain the adoption of modern educational technologies, particularly in resource-limited settings like Sudan.

Limitations

This study is limited by its reliance on self-reported survey data, which may introduce recall bias, social desirability bias, and subjectivity. Its cross-sectional design prevents assessment of changes in perceptions over time. Contextual confounders, such as the temporary relocation of the university, may have influenced responses. Additionally, individual learning styles were not assessed, which could affect engagement and knowledge retention, as reinforced by practical exercises.

Future directions

Future research should incorporate longitudinal, multi-institutional, or mixed-method designs to validate and extend these findings. Objective performance assessments, classroom observations, and experimental studies could further clarify the effectiveness of different teaching modalities and learning resources. Implementing hybrid approaches combining traditional and digital methods could address resource limitations and enhance educational outcomes.

## Conclusions

Medical students at UMST preferred a blended approach to anatomy education, combining traditional lectures, cadaveric dissection, and modern visual or digital tools. No significant gender differences were observed in preferences, challenges, or solutions. Cadaveric dissection and key textbooks remain central, but curriculum improvements, such as increased dissection hours, clinical integration, and use of 3D models or virtual simulations, can enhance learning. Limitations include reliance on self-reported data and a cross-sectional design. Future studies should use objective performance measures and longitudinal or multi-institutional approaches.

## Appendices

**Table 5:** Study questionnaire: Students’ perceptions of anatomy teaching methodologies. Adapted from [16].

Item	Question/Response options
1	Best teaching method for theory classes: a) Lecture, b) Problem-based learning (PBL), c) Small groups for discussion, d) Interactive media
2	Best viable solutions for problems in theory: a) More visual aids in lectures, b) More clinical correlation, c) Repetitive reinforcement, d) Others (specify)
3	Specific problems in understanding embryology: a) Inability to visualize, b) Inability to understand the sequence of events, c) Inadequate time, d) Others (specify)
4	Best solutions for problems in embryology: a) More time in lectures, b) Use more visual aids including 3D models, c) More time in practicals, d) Simplify information, e) Others (specify)
5	Problems in understanding histology: a) Difficult/confusing concepts, b) Lack of audio-visual aids, c) Insufficient time in lectures, d) Insufficient time in practical sessions, e) Difficult to identify structures on slides, f) Others (specify)
6	Best solutions for problems in histology: a) More time in lectures, b) Use more visual aids including 3D models, c) More time in practical, d) Simplify information, e) Others (specify)
7	Best teaching methods for tutorials: a) Chalkboard, b) Multimedia, c) Models, d) Case scenario, e) Others (specify)
8	Best multimedia method during tutorials: a) Dissection videos, b) Real pictures, c) Diagrams, d) Surgical operations, e) Others (specify)
9	Preferred methods for practical sessions: a) Dissection, b) Prosection, c) Educational videos, d) Anatomical models
10	Preferred guidance during dissection: a) Labelled structures, b) Teaching on body, c) Non-labelled structures, d) Others (specify)
11	Best solutions for practical sessions: a) More visual aids (including dissection), b) Additional time for dissection, c) Repetitive reinforcement, d) More time in tutorials, e) Others (specify)
12	Practical anatomy classes are more comprehensible when: a) Conventional teaching, b) Multimedia methods, c) Teaching with 3D models, d) Group discussion, e) Others (specify)
13	Best source of study material for anatomy: a) Teacher’s notes, b) Textbook, c) e-learning apps/websites, d) Internet videos, e) Others (specify)
14	Most used textbook: a) Snell, b) Moore, c) Gray’s, d) Last’s, e) Other (specify)
Index	Date; Gender (Male/Female)
